# A Case of Painful Menstruation, Attended with Vomiting

**Published:** 1784

**Authors:** 


III. *A Case of painful menstruation, attended with vomiting. Communicated in a letter to* Dr. Simmons, *by* Samuel Daniel, M. D. *physician at Crewkerne in Somersetshire; and extra-licentiate of the Royal College of Physicians, London.*

A Young woman, aged 19, has, ever since she first menstruated, been afflicted, at that period, with severe and incessant vomitings. They were less so, indeed, in the beginning, but for two or three years past they have regularly returned with her menses, and continued in a very severe degree during the whole time of that discharge. She says, that in a very short time after she perceives the symptoms of approaching menstruation, she is seized with a violent pain in her bowels, has a considerable tremor and a general sensation of coldness. The pain is so exquisite, that she can scarcely speak, and after a sudden, and if possible, more exquisite torture than before, she has a motion to go to stool, which is followed with a kind of syncope, and an immediate vomiting.—Between the vomitings she has a stool every quarter of an hour for four or five times. After vomiting for some time her bowels become easier, but are still in violent pain, pain, so that she cannot help crying aloud. If the vomiting begins in the morning, it continues till the next morning about the same time; sometimes longer, but always twenty-four hours, during which time she can neither eat nor sleep; and in the space of a day she is so extremely weakened, that she cannot stand without help. She has then no complaint for a month, or till the menses return. She recollects, that her menses in the beginning returned every three weeks; and were more considerable in quantity than they are at present. When the vomiting ceases she has no complaint but weakness, and after she has slept a little, and taken food she, by degrees, gets perfectly well. This complaint has continued five years, but at first it returned once in about a quarter of a year only; now it comes regularly every month. I was not ashamed to acknowledge myself at a loss with respect to the nature of this extraordinary affection; and as I was ignorant of the cause, I could not flatter myself with success in the treatment of so obscure and so inveterate a disease. It seemed probable however, that a particular degree of irritability in the bowels occasioned the circumstances above-mentioned; I therefore ordered her, upon the grounds of conjecture only, to take, take, during the interval of menſtruation, an electuary of the bark, and when ſhe perceived the pain in her bowels to be returning, about thirty drops of the *thebaic tincture* in a draught. It occurred to me afterwards, that as the quantity of the diſcharge was evidently leſſened, there would have been no impropriety in her loſing a few ounces of blood a day or two before the appearance of the menſes; this, however, as ſhe lived at a conſiderable diſtance from this place, I had no opportunity of communicating.

In about a month I received from her the following account; viz. that ſhe had taken the draught when ſhe began to grow ſick and reſtleſs as uſual, and that ſhe was ſoon afterwards compoſed and more eaſy; that ſhe ſlept, and did not riſe that day; that ſhe was not perfectly well, but did not vomit, neither was ſhe ſo ſick as ſhe uſed to be; that ſhe had taken the electuary for a week, but thought that her complaints would have been as troubleſome as ever, but for the anodyne draught.

From the time that this account was ſent to me, which was ten months ago, I had no farther intelligence till laſt week, when the gentleman from whom ſhe has her medicines informed me, that ſhe ſtill continues to uſe them, but leſs conſtantly than in the beginning; from which I infer, that the complaint ſtill ſubſiſts, but is relieved by the plan which was recommended to her.

Crewkerne,
May 4, 1784.

